# Toward Sustainability: An Overview of the Use of Green Hydrogen in the Agriculture and Livestock Sector

**DOI:** 10.3390/ani13162561

**Published:** 2023-08-08

**Authors:** Alessandra Maganza, Alice Gabetti, Paolo Pastorino, Anna Zanoli, Benedetto Sicuro, Damià Barcelò, Alberto Cesarani, Alessandro Dondo, Marino Prearo, Giuseppe Esposito

**Affiliations:** 1 The Veterinary Medical Research Institute for Piemonte, Liguria and Valle d’Aosta, 10154 Turin, Italy; alessandra.maganza@izsto.it (A.M.); alice.gabetti@izsto.it (A.G.); alessandro.dondo@izsto.it (A.D.); marino.prearo@izsto.it (M.P.); 2 Department of Life Sciences and Systems Biology, University of Turin, 10123 Turin, Italy; anna.zanoli@unito.it; 3 Department of Veterinary Sciences, University of Turin, Grugliasco, 10095 Turin, Italy; benedetto.sicuro@unito.it; 4 Institute of Environmental Assessment and Water Research, IDAEA-CSIC, C/Jordi Girona 18-26, 08034 Barcelona, Spain; dbcqam@cid.csic.es; 5 Department of Agriculture, University of Sassari, 07100 Sassari, Italy; acesarani@uniss.it

**Keywords:** aquaculture, agriculture, greenhouse gas, hydrogen technology, renewable energy, agro-livestock sector

## Abstract

**Simple Summary:**

The climate crisis we are facing increasingly requires us to achieve zero carbon dioxide (CO_2_) emissions. Green hydrogen is a gas produced from the electrolysis of water using electricity from renewable sources (e.g., photovoltaics, wind power, etc.). It represents a viable alternative to fossil fuels, without generating polluting effects.

**Abstract:**

The agro-livestock sector produces about one third of global greenhouse gas (GHG) emissions. Since more energy is needed to meet the growing demand for food and the industrial revolution in agriculture, renewable energy sources could improve access to energy resources and energy security, reduce dependence on fossil fuels, and reduce GHG emissions. Hydrogen production is a promising energy technology, but its deployment in the global energy system is lagging. Here, we analyzed the theoretical and practical application of green hydrogen generated by electrolysis of water, powered by renewable energy sources, in the agro-livestock sector. Green hydrogen is at an early stage of development in most applications, and barriers to its large-scale deployment remain. Appropriate policies and financial incentives could make it a profitable technology for the future.

## 1. Introduction

Climate change is a major challenge. The ecological disruption it causes is usually slower than that caused by other factors (e.g., land-use change, pollution, biotic exchange), with long-term effects [1] on agro-ecosystems and economic and social consequences for food security and nutrition [2,3]. Greater effects on vulnerable populations and countries can be expected in arid regions, and in landlocked and small island countries [2].

The Intergovernmental Panel on Climate Change (IPCC) identified four main areas of risk to food security: rural livelihoods and income; marine livelihoods and biodiversity; terrestrial and freshwater livelihoods and biodiversity; and food security and the breakdown of food systems [4]. Since the release of the panel’s last report, climate governance has focused on direct [greenhouse gas (GHG) limitation] and indirect (mitigation effects) climate laws [5]. Among the global initiatives in response to climate change, the 2030 Agenda for Sustainable Development is an action agenda for people, planet, and prosperity, signed in September 2015 by 193 United Nations member countries [6,7]. It incorporates 17 sustainable development goals (SDGs) and 169 targets into a grand program of action.

In 2019, China produced more than a quarter of global GHG emissions [12.7 billion tons of CO_2_ equivalent (CO_2_eq.)], accounting for 26% of global GHG emissions, three-fold higher than the 7% produced by the EU-27. These emissions (a 75% increase between 2005 and 2019) can be attributed to strong economic growth and increased energy demand. Global emissions rose by 24% during the same period, while the EU-27 reduced its emissions by 20%. China is party to the United Nations Framework Convention on Climate Change (UNFCCC), and has ratified the Paris Agreement, which has less stringent requirements. China is entitled to receive support from developed countries listed in Annex I of the Convention, because it belongs to the group of developing countries (non-Annex I) [8]. Nevertheless, it is committed to combating climate change [9,10,11,12].

The 2021 report of the U.S. National Oceanic and Atmospheric Administration (NOAA) stated that the annual greenhouse gas index (AGGI) was 1.49 (AGGI is the rate at which we are driving global warming; it has been indexed since 1990, the baseline year for the Kyoto Protocol, and the year the first IPCC Scientific Assessment of Climate Change was published), proving that direct warming caused by man-made GHGs has increased by 49% from the 1990 baseline [13].

In Europe, the EU Commission has proposed a goal of zero GHG emissions by 2050 based on a secure, sustainable, and competitive energy system (e.g., increased energy efficiency, use of renewables, end-use fuel switching, carbon capture and storage) [14,15]. With the Climate Change Act, the UK government, along with the Committee on Climate Change (CCC), has set a target to reduce GHG emissions by 100% by 2050, returning to 1990’s levels [16,17,18,19].

## 2. Agro-Livestock Sector: Greenhouse Gas Emissions and Energy Consumption

The global demand for agricultural products is increasing with the growth of the world’s population, which is estimated to reach more than 9 billion by 2050 [20]. To meet climate goals, emissions from the agribusiness sector must be reduced [21,22]. The agro-livestock sector, or agrifood supply chain, encompasses the production, processing, and distribution of food until consumption [23]. The sector generated one-third of global anthropogenic GHG emissions in three different ways: within the so-called “farm gate” via agro-livestock production activities (nearly half of total of emissions, 7.4 Gt CO_2_eq.); in pre/post-production processes (e.g., food manufacturing, retail, household consumption, food disposal, 5.6 Gt CO_2_eq.); and land-use change (e.g., deforestation, peatland drainage, 3.1 Gt CO_2_eq.) [24,25,26]. Overall, Asia and the Americas generated most of the total agro-livestock sector emissions by area and population (6.6, and 4.3 Gt CO_2_eq., respectively). Farm gate emissions were the major component of agro-livestock sector emissions in Oceania (71% of the total, or 0.2 Gt CO_2_eq.), Asia (50%, or 3.2 Gt CO_2_eq.), and the Americas (42%, or 1.8 Gt CO_2_eq.). Pre/post-production emissions were a major component in Asia (4%, or 2.9 Gt CO_2_eq.), and especially in Europe (53%, or 1.0 Gt CO_2_eq.), while land-use change-related emissions made up a major share in Africa (44%, or 1.2 Gt CO_2_eq.) and the Americas (31%, 1.3 Gt CO_2_eq.) [25], probably due to extensive agriculture and its impact on ecosystems (deforestation) on both continents.

The agribusiness sector must become sustainable to meet present and future nutritional needs and to ensure profitability, environmental sustainability, and socioeconomic equity. To this end, a strategy based on greater clean energy availability may be successful. Nonetheless, the harmful impact of fossil fuels on the environment signals the need for renewable technologies in the agro-livestock sector. Fossil fuels have been replaced with low-carbon energy sources in agriculture, demonstrating that sustainable agricultural production systems and smart energy in agribusiness may be practical and economically viable solutions to ensure energy security and achieve sustainable development. From this perspective, using renewable energy sources to meet total energy demand can improve access to energy resources, reduce energy security problems, and reduce fossil fuel dependence. The main renewable energy sources in the agro-livestock sector are photovoltaic sources, wind power, geothermal, biofuels, batteries, and energy storage systems [20].

Hydrogen production holds promise as an energy technology, but its deployment in the global energy system has been slow. Currently, few case studies have examined hydrogen energy technology, particularly that produced from renewable energy sources [27,28]. In any case, it represents a key energy resource to support and accelerate the green energy transition. Therefore, the purpose of this review is to provide an overview of the use of green hydrogen in the agriculture and livestock sector, analyzing both the pros and cons of its current and future application.

## 3. Hydrogen: A Future Energy Solution?

Hydrogen is a promising, high-energy-density, potentially clean energy carrier. One of the strategies involved in plans to transform and decarbonize the global energy system and to achieve net zero emissions by 2050 is the expansion of current hydrogen production [28]. The International Energy Agency (IEA) estimates that over the coming years, hydrogen will increase its share in total final energy consumption (TFC). In 2020, hydrogen and hydrogen-based fuels accounted for 0.1% of TFC, but this statistic is projected to see a 2 to 10% increase between 2030 and 2050 [29]. It is estimated that total annual hydrogen production will be at least 150–200 Mt by 2030, and 500 Mt by 2050 [28,29]. Between 2021 and 2050, increasing demand for hydrogen and improvements in its clean production will reduce emissions by about 60 Gt of CO_2_ (a 6.5% total cumulative emission reduction) [29].

Today, hydrogen is mainly used in the petrochemical industry to synthesize ammonia for fertilizers, for refining, and in steel and iron production; its use in other applications (e.g., transportation, shipping, heating, electricity generation) is still limited [30]. Greater use of hydrogen in the energy sector is necessary to achieve current decarbonization goals [28].

Different types of energy sources (i.e., fossil fuels, biomass, renewable energy, nuclear energy) can be used to generate hydrogen via steam methane reforming, coal gasification, cracking, pyrolysis, electrolysis, and other technologies [29,31]. The environmental impact of these technologies varies depending on the energy sources used. These impacts are also influenced by the geographical region and configuration of the production process. Different terms denote different production methods, usually with color attributes, such as gray hydrogen derived from natural gas, blue hydrogen generated from natural gas with carbon capture and storage (CCUS), and green or renewable hydrogen obtained from the electrolysis of water powered by renewable energy [32,33] (Table 1).

Color coding is not enough to define production sustainability, however. Production sustainability should be assessed, considering materials and energy requirements, environmental impact, and the readiness of technologies [34]. The IEA defines “low-carbon/low-emission hydrogen” as hydrogen generated from renewable sources, nuclear electricity, fossil fuels with CCUS, and biomass, emphasizing the pivotal role that low-carbon production pathways can play in the green energy transition [29].

In 2021, around 94 million tons of hydrogen were produced globally [28]. At least 95% of global production was obtained from fossil fuels with considerable CO_2_ emissions (roughly 900 Mt of CO_2_ per year) [28,29,33,35]. To make hydrogen part of the global process of decarbonization, its footprint has to be reduced from what it is today [28,29]. In 2021, only 1% came from renewable energy sources, and just 35 kt (0.1%) of hydrogen was obtained from electricity via water electrolysis. The amount of hydrogen produced by water electrolysis, although very small, increased by nearly 20% between 2020 and 2021, indicating the growing use of water electrolyzers [28].

Overall, hydrogen production becomes environmentally sustainable when low-carbon energy sources (solar energy, wind energy) are employed to power an electrolysis reaction. The coupling of renewable energy systems with electrolysers can supply affordable, low-emission electricity, and hydrogen [36]. The use of electricity generated from renewables like solar and wind is increasing, but the intermittency of renewables remains unresolved.

**Figure 1:**
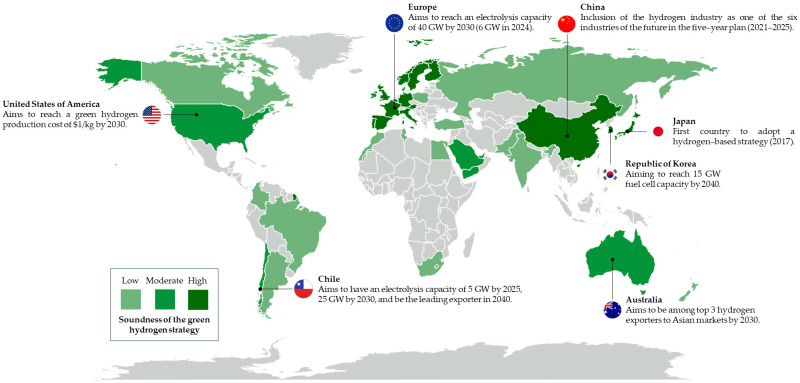
Worldwide green hydrogen production and future prospects [37].

Hydrogen can be stored and then converted to electricity when demand is high, and to offset the variable production of solar and wind power. Since electrolytic hydrogen production is affected by the cost of electricity, lowering the cost of solar and wind power could lower the price of low-emission hydrogen. Hydrogen production from renewable sources was almost zero until 2019, so it did not contribute to climate change mitigation [28,33]. Since then, the number of low-emission hydrogen production projects has grown rapidly worldwide [33]. To achieve the goal of net zero emissions by 2050, the global production of low-emission hydrogen should reach 100 million tons per year by expanding the production capacity of electrolyzers. In 2022, production capacity was 8 GW per year, although it can be expected to grow in the future (60 GW per year by 2030) [28]. By 2050, at least 60% of global hydrogen production will come from electrolyzers powered by renewable resources [29] (Figure 1).

## 4. Green Hydrogen as a Key Investment for the Energy Transition

Green hydrogen can be produced from water and electricity by an electrolyser. During electrolysis, water molecules are split into oxygen (O_2_) and hydrogen (H_2_) by passing a direct current through them, which drives the electrochemical reaction. The reaction occurs in an anode–cathode system in which water is oxidated and reduced, as described in the equation below:

$$H_{2}O\left(l\right)\to H_{2}\left(g\right)+\frac{1}{2}O_{2}\left(g\right)\;{\Delta H}_{R}^{0}=+286\;kJ/mol$$

The electrolyser uses electricity to generate hydrogen and oxygen from water. This energy can be obtained from various different sources, including renewables (i.e., photovoltaic, wind, hydropower, decarbonized grid electricity) [30]. The efficiency and the cost effectiveness of hydrogen production by water electrolysis are related to the performance, durability, and costs of the electrocatalysts used in the electrolyser. Depending on the electrolyte, operating conditions, and ionic agents (OH^−^, H^+^, O_2_^−^), four main methods of water electrolysis are distinguished: alkaline water electrolysis (AWE); proton exchange membrane water electrolysis (PEM); solid oxide electrolysis (SOE); and anion exchange membranes (AEM) [29,38]. Each technology (depending on the context) has operational and economic advantages and disadvantages [29]. Electrolyzers are usually combined with systems to store the hydrogen, which can be compressed or liquefied (in purity or in a mixture) and stored in tanks for later use. The hydrogen produced by electrolysis can be burned as fuel, generating heat without emitting CO_2_. In addition, hydrogen can be used in a fuel cell, where it chemically reacts with oxygen to generate electricity and water vapor as a by-product. The reaction produces no pollutants or GHG emissions [30]. O_2_ is produced as a by-product of electrolysis. Normally released into the atmosphere, it can also be stored or used in industrial processes (e.g., sludge and wastewater treatment, combustion, glass production, steel production) [39]. In addition, the low-temperature waste heat generated during an electrolytic reaction can be used for heating [40].

Industrial processes are not the only potential field of application for reuse of these by-products. For instance, processes in the agro-livestock sector that require high amounts of O_2_ include sewage sludge treatment, aerobic composting, and tank oxygenation on aquaculture farms [41,42,43,44]. Additionally, waste heat can be harnessed for heating greenhouses and fish tanks [40,45]. By-product recovery can help reduce the cost of hydrogen production and increase the sustainability of the entire process [40].

## 5. Hydrogen from Biomass Electrolysis: Another Possible Green Solution?

Hydrogen can also be extracted from different types of biomass, such as wastewater, sewage sludge, manure, food industry residues, and agricultural waste [46,47,48,49,50,51]. Hydrogen production from organic matter is still limited to the industrial sector due to high costs and low technological readiness [33,34]. Hydrogen is extracted from biomass by both thermochemical and biological processes (e.g., conventional gasification, pyrolysis, gasification with supercritical water, fermentation, anaerobic digestion). After conversion of the organic matter to biogas, an additional process is required to extract pure hydrogen [33]. The conversion is completed by energy, which is not necessarily a low-emissions process [52]. Coupling biomass conversion with CCUS or carbon-neutral energy sources, however, may reduce CO_2_ emissions during hydrogen production [53,54].

Recent innovative bio-electrochemical technologies, such as microbial electrolysis cell (MEC) systems, require fairly low external energy input, can use a wide range of organic materials, and can treat pollutants [34,54]. In MEC systems, electrochemically active microorganisms are used as catalysts. The organic matter is converted during an oxidation reaction that releases protons (H^+^), while hydrogen (H_2_) is produced after a reduction reaction. The electrolysis of biomass is different from electrolysis of water: in the former, the substrate is oxidized, while in the latter, oxygen gas is produced from water. Currently, MEC systems are not widely used in industry because of their slow conversion rate, and because the biomass must first be fermented before it can be converted to hydrogen by microorganisms. Future research could improve system performance and evaluate its integration into existing bio-refineries [34,53].

## 6. Hydrogen Applications in the Agro-Livestock Sector

**Figure 2:**
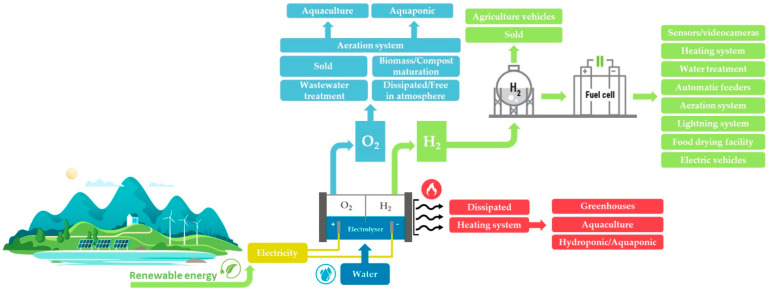
Production and use of green hydrogen and by-products in agro-livestock sector.

To date, studies and mathematical models for hydrogen applications in remote areas have described autonomous energy systems based on hydrogen production via the electrolysis of water, while electricity is generated by renewable sources [55,56,57,58]. These studies have stimulated further research into the application of this technology to the agro-livestock sector (Figure 2).

### 6.1. Literature Search

The electronic databases Google Scholar (https://scholar.google.it/ accessed on 23 May 2023) and Scopus (https://www.scopus.com/ accessed on 28 May 2023) were queried using the search terms “green hydrogen” OR “renewable hydrogen” AND “agro-livestock sector” OR “electrolysis” OR “agricultural sector” OR “polygastric” OR “monogastric” OR “poultry” OR “rabbits” OR “aquaculture” OR “aquaponic systems” OR “remote area”. From the over 100 records retrieved, only those that reported applications (including experimental applications) in the agriculture and livestock sector were selected.

### 6.2. Agricultural Sector

Experimental applications of hydrogen from renewable energy sources in agriculture have been conducted in three areas: off-road agricultural vehicles [59], greenhouses [60,61], and hydroponics [62,63]. For instance, in a vineyard in northeastern Spain, Carroquino and co-authors (2019) installed a renewable energy system. The energy generated by photovoltaic fields powered the winery’s wastewater treatment plant, irrigation system, and other auxiliary consumption [59].

Greenhouses are another of the most innovative feature of modern agriculture worldwide (especially where climatic conditions are unfavorable to plant growth). However, such systems consume huge amounts of energy [61,64]. Ganguly and co-authors (2010) modeled and analyzed an energy system that combined photovoltaics, an electrolyzer and a fuel cell applied to a greenhouse for floriculture [60]. Solutions for an energy system based on solar energy, hydrogen, and geothermal energy can be found. In this regard, Pascuzzi and co-authors (2016) built a self-sufficient experimental greenhouse consisting of an integrated system of photovoltaic panels, a water electrolyzer, fuel cells, pressurized hydrogen tanks, and a geothermal heat pump [61]. Yamaguchi and co-authors (2018) developed a small-scale hydroponic system for growing lettuce. The system is powered by renewable energy and controlled by a hybrid energy system using a hydrogen fuel cell and a lead–acid battery [62]. Swaminathan and co-authors (2022) developed and deployed an automated hydroponic system for urban agriculture that combined a solar panel, an electrolyzer, and a fuel cell. The hydrogen was generated by water electrolysis fed by the solar panel during daylight, then stored for use by the fuel cell in the absence of sunlight. The residual heat was used to heat the plants [63].

### 6.3. Livestock Sector

Though studies on low-emission hydrogen production from biogas and animal waste have been conducted [65,66,67,68,69], very little research has gone into the potential use of hydrogen from electrolysis to meet the energy demand of livestock farms.

#### 6.3.1. Polygastric and Monogastric Animals

There are no studies on the use of hydrogen on monogastric animal farms, whereas pilot studies have been carried out involving polygastric animals.

One example is the “HydroGlen” project conducted by the James Hutton Institute in Scotland. The project aims to transform the Institute’s research farm into a self-contained, low-emission facility powered by green hydrogen that can provide energy to the local farming community, thus demonstrating how the farming community can play a major role in Scotland’s decarbonization plans through green hydrogen production and use. In the feasibility study, energy requirements (i.e., heating, lighting, transportation) were calculated and expressed in kilowatt hours (kWh). This information was then used for modeling the software that ran energy generation and storage scenarios and determined the size and design of the hydrogen plant components. The generation plant consists of renewable energy generators (solar panels, wind turbines), a grid connection, a water demineralization system, an electrolyzer, a battery, a hydrogen compressor, hydrogen storage facilities, a hydrogen vehicle fueling station, hydrogen fuel cells, and electric vehicle charging stations [70].

#### 6.3.2. Poultry and Rabbits

Few studies have investigated the costs and benefits of using low-emission hydrogen as an energy source on poultry farms. One of the first studies to examine the use of hydrogen generated from renewable sources on a farm was published in 1981 [71]. The study analyzed a hypothetical broiler farm in which the heating and ventilation systems were powered by hydrogen produced by an electrolyzer. The energy for the electrolytic reaction came from a windmill, making the entire system independent of fossil fuel.

More recently, Genç and co-authors (2012) analyzed the costs of a wind–electrolyzer–fuel cell system in the Kayseri region of Turkey. The area has wind potential and is an important part of the economy based on livestock farming. The study described a hypothetical grid-dependent system consisting of a wind turbine, an electrolyzer, and a fuel cell. Daily and annual energy consumption (kWh/d, MWh/y) of the lighting, air conditioning, and feed handling systems were calculated to estimate the amounts of hydrogen and electricity needed, as well as the production costs. The study showed that hydrogen production costs are closely related to the rated power of the electrolyzer, the cost of electricity generated by the turbine, and the height of the turbine hub [72].

Currently, there are no studies on the use of hydrogen on rabbit farms.

#### 6.3.3. Aquaculture and Aquaponic Systems

Several private companies are experimenting with the use of hydrogen in aquaculture. Most of the few studies published to date report theoretical applications and cost–benefit assessments based on mathematical models. The use of green hydrogen as an energy source in aquaculture could provide for better utilization of solar and wind energy throughout the year. The combination of fuel cells and electrolyzers powered by renewable energy can provide a backup energy source with low emissions and greater flexibility. In addition, it could make off-shore systems located in less accessible areas more energy-independent [73].

Oxygen, the by-product of electrolysis, holds interest for aquaculture. Maintenance of water quality is one of the major goals in aquaculture. One of the critical parameters to ensure animal health and survivability is the concentration of dissolved oxygen. Electrolytic oxygen can be directly employed in the aeration system, especially in intensive aquaculture systems of aquatic species needing dissolved oxygen and reared in high production densities [43]. The oxygen generated by electrolysis could partially compensate aeration costs, reduce energy demand, and raise production yield [41].

In southwest Spain, the AQUASEF project (LIFE13 ENV/ES/000420) applied the idea of self-generated oxygen from renewable energy sources (wind turbines, photovoltaic panels) on aquaculture farms. Pure oxygen produced by electrolyzers is used for enhancing aeration in some key stages of the breeding process. Stored hydrogen is recycled for power generation by the fuel cell system. The fuel cells are designed for use in a backup system to ensure power supply at the facility. Overall, CO_2_ emissions were reduced (at about 35 tons per year equivalent), and about 65 MWh per year was generated from renewable energy sources. Additionally, a considerable amount of oxygen was produced on site (6.7 tons per year) and employed in the aeration system, resulting in an 80% reduction in oxygen consumption [74].

The Mekong Delta (Vietnam) has abundant renewable energy sources. The use of hydrogen has been investigated and applied on a shrimp farm [41]. The system was composed of shrimp ponds, wind turbines, photovoltaic arrays, batteries, an electrolyzer, proton-exchange membrane (PEM) fuel cells, storage systems for oxygen and hydrogen, a microbubble generation system, and a water treatment system. Wind turbines and photovoltaic arrays during daylight generate electric power for the water treatment system and the other facilities on the farm. The surplus power is stored in a battery and then used by an alkaline electrolyzer to generate pure oxygen. Both the water treatment system and the electrolyzer run on a stable flow of electricity to supply clean oxygenated water for the shrimp ponds. For this reason, the battery continuously feeds the electrolyzer to ensure stable oxygen production [43]. Pure oxygen is pumped through pipelines at the bottom of the shrimp ponds. Replacing air (21% of O_2_) with pure oxygen reduces the volume of gas injected into the ponds and the energy needed for the compressors by a factor of five [75]. The hydrogen produced by electrolysis is used to regenerate electricity by the fuel cells and sold to the national electric grid, but it could also be used as a load-leveling electrical system [57]. Hydrogen is more efficient than fossil fuels in converting electricity into other useful forms of energy [76].

As part of a master’s thesis project at the University of Stavanger in 2022, Røstbø and Torgersen studied the opportunities and challenges of combining water electrolysis systems with recirculating aquaculture systems for Atlantic salmon (*Salmo salar*). The authors described three different case studies of such facilities of increasing size and evaluated, using mathematical simulation, the technical feasibility, the energy demands, and the production cost of hydrogen. In the models, the produced oxygen was used instead of air in the aeration system, and the surplus was sold [77].

Janke and co-authors (2020) evaluated the techno-economic feasibility of a small hydrogen production plant installed on a cereal farm in Sweden. The aim was to determine whether the on-site production of hydrogen could meet the fuel demand of farm tractors and vehicles. Based on mathematical modeling, the authors described different scenarios of electrolytic hydrogen production from renewable energy (wind), and evaluated the initial investment, annual costs, total production of hydrogen, oxygen, and waste heat, and carbon abatement costs. The by-products of the electrolysis reaction were valorized: waste heat was integrated into the heating system of a greenhouse for intensive tomato cultivation, while oxygen was integrated into the aeration system of a tank for rainbow trout cultivation. The assessment demonstrated that the installation of hydrogen plants powered by wind energy could be effective in the decarbonization of agricultural systems [40].

Mohammadpour and co-authors (2021) described a strategy to cut the costs of production and storage of electrolytic hydrogen through the use of by-product oxygen. They demonstrated how oxygen could be successfully used in the aeration of wastewater in sludge treatment plants or in the aquaculture industry. A model was designed to evaluate the advantages of using pure oxygen instead of air in a wastewater treatment plant; the study demonstrated how using oxygen as a by-product could offset the energy cost for hydrogen storage by up to 30%. The study also showed that savings up to 60% in energy could be achieved when oxygen was employed in aquaculture to aerate fish or shrimp tanks. The authors reported that the higher the oxygen requirement of a process, the higher the savings gained from using pure oxygen instead of air. The use of oxygen generated from on-site electrolysis can help make the aeration system more sustainable [42].

The use of hydrogen in offshore facilities presents both opportunities and challenges. It could meet the energy demand of lights, cameras, and sensors, while providing back-up power for aerators and food dispensers. Oxygen produced locally during electrolysis could be used to aerate and potentially reduce sludge. Hydrogen could reduce CO_2_ emissions and costs and aid in more sustainable food production. A major point is that hydrogen integration requires high initial investment, specific plant and storage system design, and potential operational difficulties to overcome [73].

To promote the sustainable exploitation of marine resources, the European Union funded the H_2_Ocean project in 2012 to develop and design a multi-component, multi-purpose offshore platform equipped with a hydrogen generation plant powered by renewable energy (wind, waves), a multi-species aquaculture farm, and environmental monitoring systems. The design was intended to be flexible, so that it could be adapted to different locations and economies. The environmental and economic impact was assessed. In the experimental design, hydrogen was generated from desalinized seawater using an alkaline electrolyzer powered by renewables, and then stored for later use or for transport and sale. The oxygen derived from electrolysis was stored and used to improve fish growth and to prevent algal blooms. The aquaculture farm reared fish from different trophic levels in combination with mollusks and sea urchin cultures (which used the effluents from the fish cages); the multi trophic aquaculture reduced the pressure on wild pelagic fish. Completed in 2014, the H_2_Ocean project showed that the platform could be a good alternative provider of sustainable food and clean energy [78].

Hydrogen can be produced by saline water electrolysis, powered by renewable energies; it can be obtained directly from seawater, or indirectly after desalination. One method has no electrode side reactions or corrosion issues, but requires additional energy, while the other method is not yet widely used, but has high potential for hydrogen generation [79]. As freshwater becomes scarcer for many communities, saline and impure water is still an abundant resource; future research could be aimed at developing new electrode materials and membranes capable of functioning with saline and low-grade water [80].

As part of a master’s thesis project at the University of Bergen in 2022, Sandøy conducted a feasibility study of a zero-emission offshore fish farm powered by wind power and electrolytic hydrogen in Norway. The study comprised two case studies that included just wind power and hydrogen in the first case, and an added diesel generator in the second. Both cases had a simulated duration of 20 years. The power demand of the farm, the technical characteristics of the turbines, the electrolyzer, the desalinator, the hydrogen storage capacity, and the costs were described in both cases. The author concluded that the first scenario, wherein the energy system consisted of wind power and hydrogen, was unprofitable. The second scenario was found to be more profitable, because the diesel generator decreased the need for hydrogen storage and wind power capacity. Based on the simulations, the addition of a diesel generator to the system reduced the CO_2_ that would have been released if only diesel had been used by over 80% [81].

Aristokleous and co-authors (2022) analyzed the energy demand of a model offshore aquaculture farm in the Mediterranean (with an annual production of 2000 tons of fish), and hypothesized the use of hydrogen from renewables as the main fuel to power the farm. The energy needed to sustain the facility (outdoor lights, surveillance cameras, sensors) was calculated and expressed as kilowatt hours per day (kWh/day). To meet the energy demand, various different solutions were compared: a combination of a hydrogen fuel cell and an electrolyzer; a system consisting of solar panels, a water purifier, and an electrolyzer; and lastly, a floating photovoltaic system. It was concluded that the two hydrogen solutions were favorable, because at the same cost, they had fewer disadvantages than the photovoltaic system [82].

**Figure 3:**
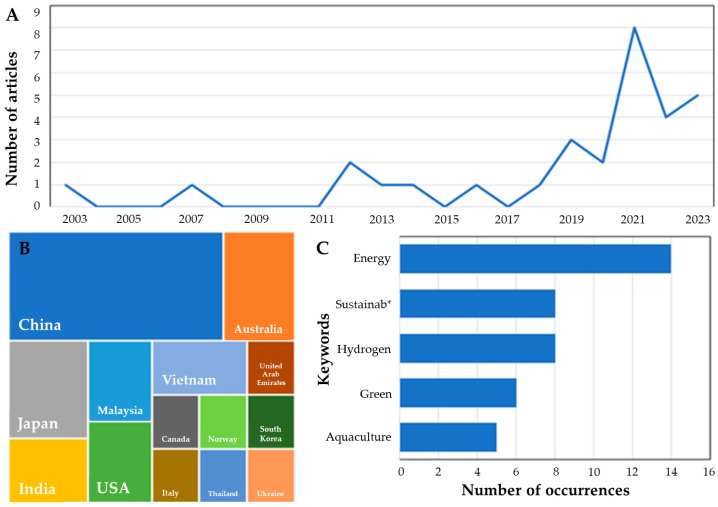
Scientific articles published on aquaculture and hydrogen utilization, following a text mining approach. (**A**) trend of articles; (**B**) geographic distribution of research; (**C**) most popular keywords utilized by authors. (sustainab* corresponds to all the words starting with same term).

In order to give a general idea about the use of hydrogen in aquaculture farms, a quantitative analysis of published articles was carried out by consulting the main databases, i.e., SCOPUS and Web of Science (WoS), using a text mining approach [83]. From 150 papers initially selected, 31 articles were found to be strictly related to the subject, with a clear increase in interest in 2021 (Figure 3A), particularly in China (Figure 3B), which was the first aquaculture producer country in the world. The keywords used in the selected articles (Figure 3C) confirm that the scientific community involved in these pieces of research on hydrogen utilization in aquaculture imagine these studies within the framework of sustainable development.

## 7. Remote Area Applications

Hydrogen as an energy storage system is a valuable resource for off-grid areas, remote islands, and for locations where technologies are needed to manage the intermittency of local renewable energy sources [84]. Temiz and Dincer (2022) designed an innovative system consisting of an ocean thermal energy conversion, a solar and photovoltaic plant, a desalinator, a PEM electrolyser, a heat pump, and a fuel cell. The system was designed for the generation, storage, and utilization of low-emission hydrogen, with the goal of sustaining the production of energy, fuel, heat, food, and freshwater for remote arctic communities in northern Canada. The food production system included a greenhouse, a fish farm, and a food drying facility. This type of research aims to reduce the diesel fuel dependence of isolated arctic settlements, which are particularly affected by climate change and food scarcity. The authors used several methods and software models to analyze the system’s energy demand, to quantify fuel, electricity and food production, and estimate the annual costs. They concluded that the system could potentially produce an average of 283 tons of hydrogen, 402 tons of freshwater, 7.9 tons of vegetables, and 374 tons of fish per year [45].

## 8. Outlook and Conclusions

Green hydrogen is a key energy resource to support and accelerate the energy transition, with global targets already identified between 2030 and 2040. The process of energy transition is not new in history. What distinguishes this transition from the previous one is the urgency of protecting the planet from climate change. Therefore, it is essential to create a sustainable food system that does not damage people and the environment.

Beneficial management practices in crop and animal production systems (i.e., manure management, precision feeding techniques, diet adjustment, optimization of grazing systems, improved genetic selection) based on agroecology studies could provide opportunities for reducing GHG emissions [85,86,87,88]. Furthermore, farms integrated with renewable energy sources are both reliable and efficient in reducing fossil fuel consumption and carbon emissions [20]. Solar, wind, geothermal, and other renewable energy sources are used for producing hydrogen by water electrolysis. Hydrogen promises to be the most interesting alternative to fossil fuels, as both energy transport and storage, solving the problem of the intermittent availability of renewable energy [89].

This review presents various different applications of water electrolysis in the agro-livestock sector, but green hydrogen could also be applied to the decarbonization of the marine industry, using offshore wind energy to provide clean fuel [89]. Moreover, water electrolysis powered by renewable energy sources and other systems can be further improved to produce hydrogen sustainably. The agro-livestock sector could benefit from the bio-electrochemical production of hydrogen from low-value biomass, since agricultural waste and livestock manure are an abundant and renewable source of energy [90]. Future research should develop promising technologies, such as MEC, or other biological processes [34,47]. This could be convenient from the perspective of a circular economy.

Green hydrogen is currently at an early stage of development, and barriers to its wider application persist. Production can cost up to EUR 15 per kg [91] more than hydrogen generated from fossil fuels without CCUS [28]. The potential value of green hydrogen is still underappreciated; for example, it could ensure energy security by diminishing fossil fuel dependency. Finally, much of the current infrastructure is still at a low technology readiness level. Many production plant projects are in the planning phase, and few have reached the decision stage for final investment, largely due to uncertainties surrounding demand, the availability of sufficient electricity generation capacity, and lack of guidelines [28]. Appropriate policies and financial incentives are needed to overcome these barriers and to reduce the production of hydrogen from fossil fuels [30].

Green hydrogen production occurs through the process of water electrolysis, which requires the use of renewable energy to power the electrolyzer. This allows us to store the excess energy produced by renewables instead of wasting it. Therefore, green hydrogen represents a promising solution for the transition to clean energy, thereby solving the problem of the intermittent availability of renewable energy due to its renewable nature and its ability to be used in various sectors, including the agro-livestock sector. There are still challenges some to overcome, but continued technological advances show that this will be an increasingly important component of the clean energy of the future.

## Figures and Tables

**Table 1:** Different shades of the main hydrogen types [30,33].

Color	Grey Hydrogen	Blue Hydrogen	Turquoise Hydrogen *	Green Hydrogen
**Process**	SMR or gasification	SMR or gasification with carbon capture (85–95%)	Pyrolysis	Electrolysis
**Source**	Methane or coal	Methane or coal	Methane	Renewable electricity
**CO_2_ emissions**	9–20 kg of CO_2_ generated per kg of product	1.5–4.5 kg of CO_2_ generated per kg of product	0 kg of CO_2_ generated per kg of product	0 kg of CO_2_ generated per kg of product
**Benefits**	Low production cost	Use of existing assets via carbon capture and storage, with lower GHG emissions	No CO_2_ produced	Consistent with net zero CO_2_ emissions
**Disadvantages**	High CO_2_ emission makes these technologies unsuitable for a sustainable pathway (net zero emission)	Carbon capture can never be 100% efficient as it is subject to fossil fuel availability and price fluctuations and does not meet the criteria of a net zero future	Still at pilot stage, no industrial applications, the carbon in the methane turns into solid carbon black material	Infrastructure, policies, value recognition currently lacking; production cost is 2–3 time higher than gray hydrogen
**Estimated % of respect for the environment**	0%	33%	66%	100%

SMR = steam methane reforming; * Turquoise hydrogen is an emerging decarbonisation option.

## Data Availability

Not applicable.
